# Sustained Antibiotic-Eluting Intra-Ocular Lenses: A New Approach

**DOI:** 10.1371/journal.pone.0163857

**Published:** 2016-10-14

**Authors:** Dulcia W. N. Tan, Soo Ghim Lim, Tina T. Wong, Subbu S. Venkatraman

**Affiliations:** 1 School of Materials Science and Engineering, Nanyang Technological University, Singapore, Singapore; 2 Singapore National Eye Centre, Singapore, Singapore; 3 Singapore Eye Research Institute, Singapore, Singapore; 4 Ocular Therapeutic Engineering Centre, Nanyang Technological University, Singapore, Singapore; Helsingin Yliopisto, FINLAND

## Abstract

Currently, infections following cataract surgery are not as effectively managed with antibiotic eye drops, which suffer from poor bioavailability of drug and low patient compliance. The ideal solution, which can help to overcome the issue of drug wastage and poor bioavailabilty, as well as the need for frequent applications (patient inconvenience), is a drug-eluting intraocular lens (IOL). We describe a novel approach to such a drug-eluting lens by using a peripheral IOL attachment as a drug depot to deliver antibiotics, Levofloxacin (LFX) or Moxifloxacin (MFX). In this work, drug was entrapped within a fully-degradable polymer, poly(L-lactide-co-ɛ-caprolactone) (PLC). The effects of drug loading and solvent type on drug release and film morphology were investigated using cast films. The study clearly demonstrated that a slower-evaporating solvent tetrahydrofuran (THF) resulted in a better surface morphology, as well as lower initial burst compared to dichloromethane (DCM), and hence, was better suited to developing a drug-eluting attachment with sustained release of drug. When attachments were fabricated with drugs at high loading percentages (20% and 25% in polymer), significant burst was observed compared to films: this is attributed to the higher surface-to-volume ratio of the attachments. When the levofloxacin (LFX) loading percentage was decreased to 3% and 5%, the attachments presented lower burst and sustained release with therapeutic efficacy. This work has demonstrated the potential of using an IOL attachment as a more efficacious anti-infective option compared to daily eye drops.

## Introduction

Cataract is a leading cause of visual impairment globally with its prevalence on the rise annually. To date, it accounts for 51% of blindness world over, and the number of cataract operations is estimated to increase to 32 million by 2020 [1]. Cataract is treatable by surgical removal of the cloudy lens and replacing with an artificial synthetic intraocular lens (IOL). Although IOL implantation is highly successful, the potential risk of severe postoperative infection namely, Endophthalmitis is ever present [2, 3]. If left untreated, this could lead to permanent blindness. In developing countries, this is highly evident due to poor accessibility and affordability to medication.

Current postoperative management is a two week prophylactic treatment of topical antibiotics against bacterial infections such as acute endophthalmitis [4, 5]. The drawback of this treatment regimen is its poor bioavailability (5%) due to poor drug penetration into ocular tissues [6]. Permeability barriers and rapid nasolacrimal drainage contribute to low drug efficacy [7, 8]. To overcome this, daily frequent dosing is required but this result in poor patient compliance and even improper administration, especially by elderly patients. The ineffectiveness of this delivery method is its Achilles heel.

Over the years, there have been many attempts to develop IOL as a drug delivery system, such as drug-soaked IOLs [9–13], insertion of hydrogel attachments [14, 15] or drug inserts to the haptics [16–18]. Soaking IOL in drug solution is cost effective but this method suffers from short delivery period as well as deficient drug loading and drug wastage. The implementation of drug inserts to the IOL can overcome these problems to an extent, by increasing the release duration and providing better drug efficacy. However, insert attachments to the fragile haptics post a challenge for deployment, and there is also potential risk of post-surgical IOL decentralization if disorientation of the inserts occurs.

**Fig 1:**
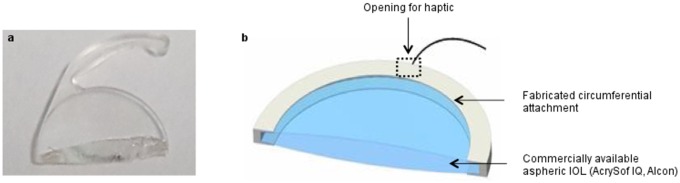
(a) Photograph of a commercial IOL cross-section and (b) schematic diagram of proposed peripheral drug-eluting attachment to a commercial IOL.

An ideal sustained drug delivery approach would be to combine treatment and surgical implantation in a single procedure. Our approach is to apply a fully biodegradable drug-containing peripheral attachment to the IOL without obstructing vision. Hook structures were designed to allow the attachment to band circumferentially around the IOL and 2 haptic holes to accommodate the IOL haptics (Fig 1). This design is to minimize accidental dislodgement. The attachment is produced using ultrasonic spray coating. The coating process moves the IOL conjugate-designed mandrel translationally and rotationally towards the drug-polymer spraying stream. The mandrel is coated for a predetermined set of loops to achieve optimized uniform thickness. The integration of drug delivery system with IOL enables localized and sustained drug release at the immediate vicinity of the device without the need for postoperative management.

Fluoroquinolones are a family of synthetic antibiotics with potent and broad-spectrum bacterial activities against numerous pathogens such as Staphylococci [19, 20]. Levofloxacin (LFX) and Moxifloxacin (MFX), which are commonly used fluoroquinolones were chosen as the therapeutic agent in this study. LFX, the L-isomer of racemic Ofloxacin (OFX) demonstrated greater corneal penetration into aqueous humor than OFX [21]. A single dose of 0.5% ophthalmic solution demonstrated high bioavailability in the tear fluid for at least 6 hours, with concentration attained above the minimum inhibitory concentration (MIC_90_) of most pathogens [22]. Growing bacterial resistance to LFX and its predecessors, led to the development of the 4^th^-generation antibiotic MFX. Structural changes in the fourth generation leads to greater potency against Gram-positive bacteria while retaining the spectrum activity against Gram-negative bacteria. The 8-methoxy structure of MFX makes it a hydrophobic fluoroquinolone which is resilient to bacterial cells’ efflux mechanism; thus, enhancing its potency against bacteria [23, 24]. It has also been shown to have better penetration into the anterior chamber with higher corneal, aqueous and vitreous concentrations achieved [25, 26].

Biodegradable poly(L-lactide-co-ɛ-caprolactone) (PLC) was selected as the polymer matrix for drug encapsulation because of its biocompatibility and flexibility [27–29]. In this paper, we report the characterization of formulation parameters, i.e. the hydrophilicity of films, effect of drug loading and solvent on the drug release performance, followed by integration into the prototype IOL attachment.

## Materials and Methods

### Materials

Poly(L-lactide-co-ɛ-caprolactone) (PLC, molar ratio 70/30, Mw = 202,000 g/mol) was purchased from Corbion Purac, The Netherlands. Levofloxacin and Moxifloxacin were purchased from Molekula Limited, UK. Phosphate buffer saline tablets (PBS) were purchased from Sigma-Aldrich, Singapore. HPLC grade dichloromethane (DCM) and tetrahydrofuran (THF) were obtained from Tedia, USA.

### Film Preparation

The drug-polymer solution was prepared by dissolving LFX or MFX (15 wt%, 20 wt% and 25 wt%) in different solvents, Dicholoromethane (DCM) and Tetrahydrofuran (THF) before adding PLC pellets (0.20 g/mL). Solutions were mixed and solvent casted to form multi-layered films. Thermogravimetric analyzer (TGA 2950, TA Instruments) was used to validate the solvent residual in the films (< 1 wt%). The thicknesses of fabricated films were 0.22 ± 0.02 mm (Elcometer 456, Elcometer (Asia) Pte Ltd).

### Contact Angle Measurement

Contact angle was measured using First Ten Angstrom, FTA32. Film-coated slides were positioned under the mechanical actuated syringe plunger. Contact angle was measured immediately after a water [solvent] droplet has detached from the needle tip and landed on the substrate.

### Surface Morphology

Morphological observation was carried out with scanning electron microscopy (SEM) Jeol JSM 6360 and JSM 5410 (Jeol USA, Inc) at 5 kV. Films were gold sputtered for 60s and attachments were sputtered for 30s.

### IOL Attachment Fabrication

**Fig 2:**
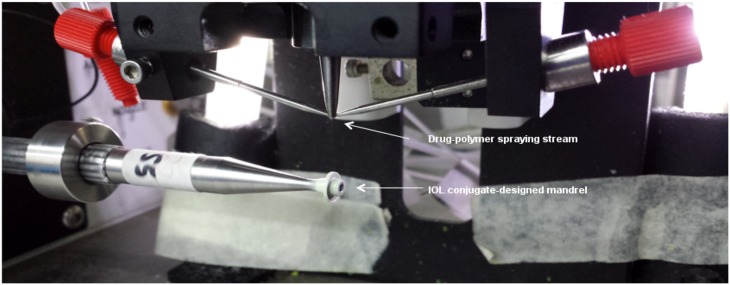
Spray coating process that moves the IOL conjugate-designed mandrel towards the drug-polymer stream.

Medicoat DES1000 (Sono-tek Corporation, USA) was used to spray coat the drug-polymer solution onto an IOL conjugate-designed mandrel to achieve the desired drug-eluting attachment embodiment (Fig 2). The spraying flow rate was set at 0.05 ml/min with a rotational speed of 40rev/min and a translational distance of 0.4 cm. The coated mandrels were left to dry in ambient condition overnight and transferred to a 37°C vacuum oven for a week.

### *In Vitro* Release Study

Triplicate film samples (1 x 1 cm^2^) were immersed in PBS and placed in 37°C incubator and sink conditions were maintained throughout. The release study was carried out for 2 weeks. At each predetermined time point, samples were transferred to fresh medium. Aliquots from the collected buffer solutions were tested for drug amounts using the fluorescence light detector (FLD) of high performance liquid chromatography Agilent 1100 series (HPLC, Agilent Technologies, USA). IOL attachment samples were prepared in triplicate and amount of drug released was detected using the same method described.

### Statistical Analysis

Experiments were carried out in triplicates. Results were presented as mean ± SD, unless otherwise stated. Statistical analysis was performed using GraphPad Prism 5.0.

## Results

### Hydrophilicity of Drug-loaded Films

**Fig 3:**
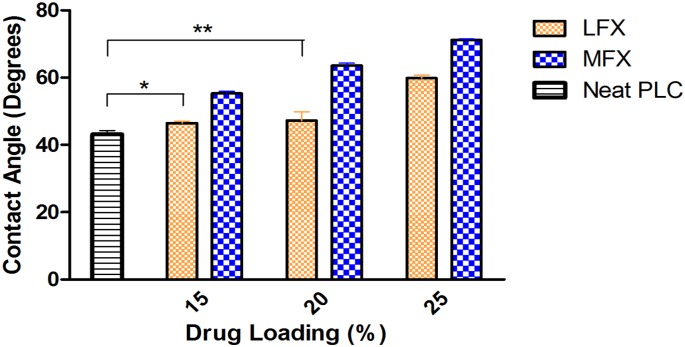
Hydrophilicity comparison of PLC-LFX, PLC-MFX and neat PLC films using contact angle measurement.

The effect of LFX and MFX on the hydrophilicity of the IOL attachment was evaluated with different drug concentrations. Surface hydrophilicity of drug-loaded films was quantified using contact angle measurement. Fig 3 shows the hydrophilicity of neat PLC film, LFX-loaded films (PLC-LFX) and MFX-loaded films (PLC-MFX) of different drug loadings. As hydrophilicity increases, the contact angle of the water droplet on the surface will decrease. From the contact angle result, three key observations can be made. Firstly, the addition of drugs into PLC matrix increased the overall hydrophobicity of the drug-polymer matrix. Secondly, for both drugs, the matrix hydrophobicity increases with increasing drug concentration. Thirdly, LFX-polymer matrix is more hydrophilic than MFX-polymer matrix. The contact angle measurements indicated the hydrophilicity difference of the two drugs and the effect of their respective hydrophilicities on the release performance will be explained in the later section.

### Morphological Analysis

**Fig 4:**
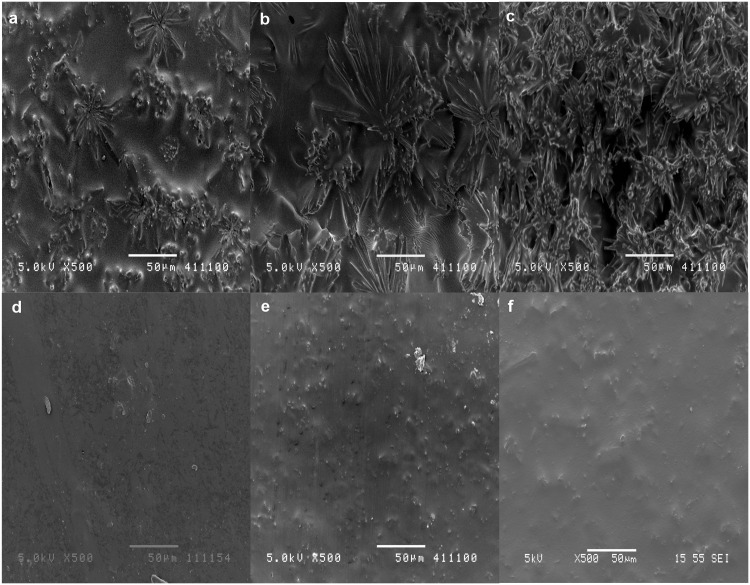
Surface morphology of DCM films. SEM micrographs of 15%, 20% and 25% LFX loaded films (a, b, c) and 15%, 20% and 25% MFX loaded films (d, e, f).

Surface morphological examination is another factor that could affect the release behavior, i.e. films with greater porosity result in higher burst. Thus, the surface morphologies of drug-loaded films fabricated in DCM and THF were investigated. Films used were 99% dried before conducting morphological analysis and release experiments. Fig 4 illustrates the SEM micrographs of 15 wt%, 20 wt% and 25 wt% LFX (a-c) and MFX (d-f) loaded films fabricated using DCM as the solvent. From the images, greater porosity could be observed in LFX films compared to MFX films and within the matrix of LFX, porosity increases with increased drug loading.

**Fig 5:**
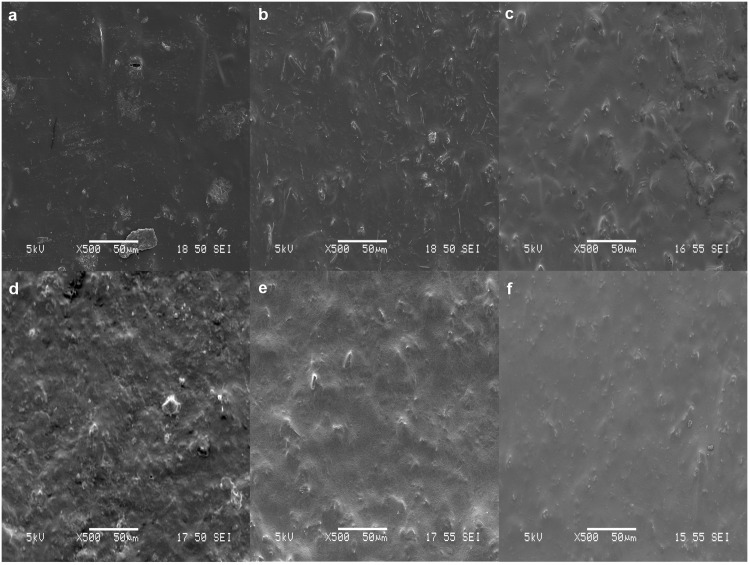
Surface morphology of THF films. SEM micrographs of 15%, 20% and 25% LFX loaded films (a, b, c) and 15%, 20% and 25% MFX loaded films (d, e, f).

The SEM micrographs of LFX and MFX (using THF as the solvent) are presented in Fig 5. A solvent change showed LFX-THF films having less porous surfaces compared to LFX-DCM. On the other hand, surface morphologies of MFX-DCM (Fig 4d–4f) and MFX-THF films (Fig 5d–5f) were similar with the exception of lesser drug aggregation on MFX-DCM films.

### *In Vitro* Drug Release

**Fig 6:**
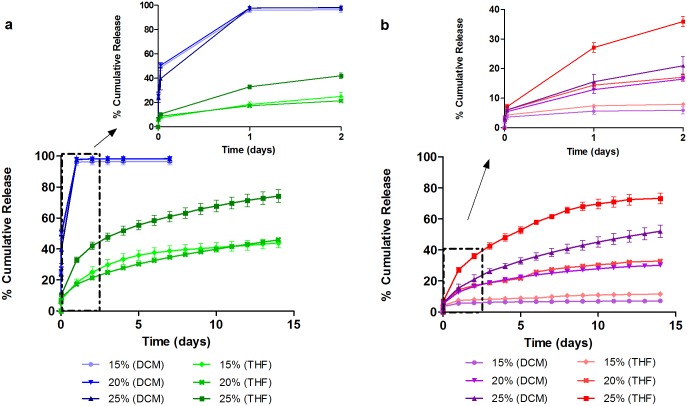
Effect of drug loading and solvents on cumulative release profiles of (a) LFX and (b) MFX. All release sustained for 14 days except for LFX-DCM films, where most drug were unloaded during initial burst (within 24 h).

The effect of drug loading and solvents on the drug release performance of LFX and MFX was investigated for 2 weeks. Fig 6 illustrates the release profile of LFX and MFX films fabricated using DCM and THF. All LFX-DCM formulations unloaded more than 95% of the drug reservoir during the initial burst release phase (within the first 24 hours), giving a release of 96.0 ± 2.1% for 15wt%, 97.8 ± 2.4% for 20wt% and 97.7 ± 0.4% for 25wt% drug loading. No significant drug was detected over the next few days suggested that the amount of drug released could be lower than the detection limit and hence, ending the study prematurely. A solvent change to THF showed suppressed burst and sustained release for LFX films to the end of the 14-day release study. 15% LFX-THF film exhibited an initial release of 18.5 ± 2.1%, while 20% and 25% film had an initial burst of 17.3 ± 0.7% and 33.0 ± 1.7% respectively. At the end of the study, 15%, 20% and 25% achieved a respective final release of 43.8 ± 3.0%, 45.9 ± 0.9% and 74.2 ± 4.2% (Fig 6a). The release observed is in view of the surface morphology of the films, whereby porous surfaces lead to high initial burst and short release duration.

In the case for MFX, both solvent systems showed an increasing trend of initial burst and release when drug loadings were increased. 15% MFX-DCM film exhibited an initial burst of 5.6 ± 1.1% (Fig 6b). When loading was increased to 20% and 25%, the initial burst increased to 12.9 ± 1.3% and 15.5 ± 2.5%, respectively. At day 14, the films each achieved a final release of 7.1 ± 1.1%, 30.2 ± 0.8% and 52.0 ± 4.0%. In comparison, an initial burst of 7.4 ± 0.3%, 14.4 ± 0.9% and 27.1 ± 1.6% was observed for 15%, 20% and 25% MFX-THF films. The final releases obtained from the films were 11.5 ± 0.6%, 32.8 ± 1.3% and 73.3 ± 3.4%, respectively. Based on these data, THF was chosen as optimal solvent for the fabrication of IOL attachments. PLC undergoes degradation in 6 months [30] and a 100% release would be expected when the polymer fully degrades.

### IOL Attachment Fabrication

**Fig 7:**
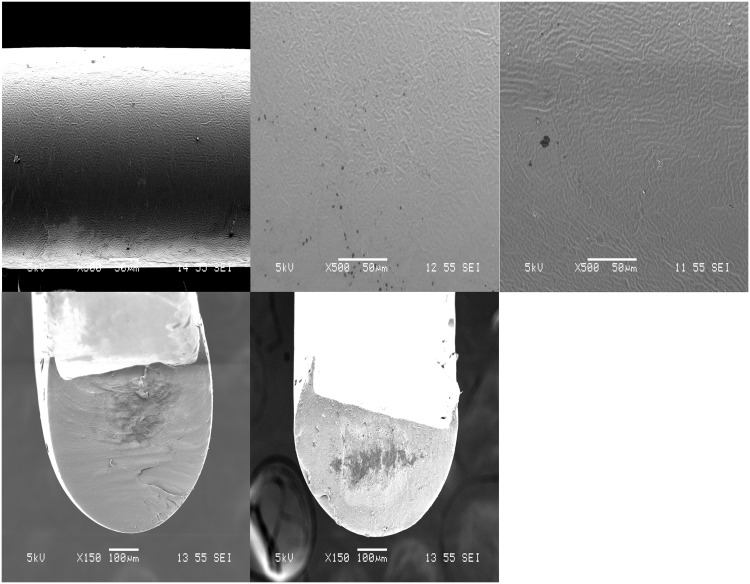
Surface morphologies of IOL attachment. SEM images of (a) neat PLC, (b) LFX loaded PLC attachment, (c) MFX loaded PLC attachment. Cross section images of attachment with hook structures: (d) PLC-LFX and (e) PLC-MFX.

Fig 7a–7c shows the SEM surface morphologies of coated attachments from PLC and formulations with LFX and MFX respectively. All formulations had smooth and uniform surface coatings. Fig 7d and 7e show the cross section of the attachment loaded with LFX and MFX, respectively. Hook structures shown in the cross section images were necessary to secure the attachment along the circumference of the IOL.

### IOL Attachments *In Vitro* Release

**Fig 8:**
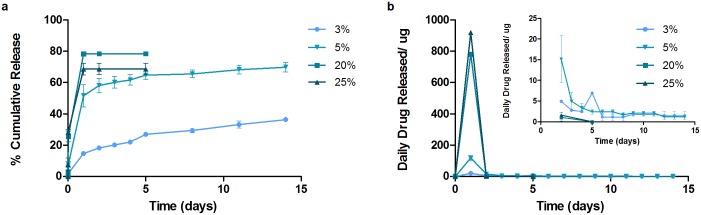
Cumulative release and daily release profiles from LFX attachments. High loaded attachments (20% and 25%) exhibited high initial burst that released most drug in 24 h. Only 3% and 5% sustained for 14 days.

Fig 8 plots the cumulative drug release of LFX from attachments. It is notable that for 20% and 25%, a higher initial burst was observed (as compared to the films) with most of the drug within the depot released. The initial burst of 20% and 25% were 78.4 ± 1.0% and 68.6 ± 3.8%, respectively. A second study was conducted with lower drug concentration. The 3% drug-loaded attachment portrayed a 14.7 ± 0.7% initial release for the first 24 hour, whereas the initial release of the 5% attachment was significantly higher at 51.6 ± 7.1%. Only 3% and 5% formulations showed sustained release for 14 days, achieving a final release of 36.4 ± 0.1% and 69.9 ± 3.0%. The daily drug release from 3% and 5% was above the required therapeutic dosage with respective total average of 50 μg and 161 μg.

**Fig 9:**
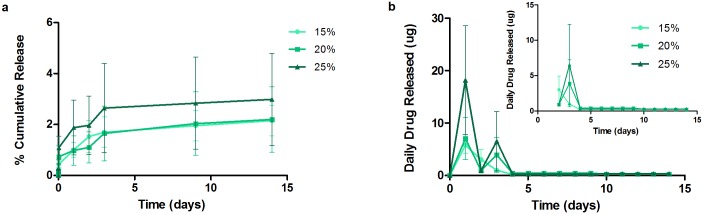
Cumulative release and daily release profiles from MFX attachments. All attachments suffered from drug exhibited low drug release.

Fig 9 illustrates the release profiles of MFX coatings and films. The initial burst observed for 15%, 20% and 25% were 1.0 ± 0.3%, 1.0 ± 0.6% and 1.9 ± 1.1%. A plateau behavior was observed after day 3. The final releases achieved from the films were 2.2 ± 0.6%, 2.2 ± 1.2% and 3.0 ± 1.8% respectively.

## Discussion

This work sought to develop a novel drug-eluting attachment to eliminate topical eye drops. Two antibiotics, LFX and MFX were utilized in this study and in the first phase of formulation characterization, the hydrophilicity of the films was studied. The changes in hydrophilicity with drug and different concentrations were investigated. Contact angle measurements obtained showed higher contact angles for MFX film. This affirms that MFX-PLC is more hydrophobic than LFX-PLC. Upon addition of LFX and MFX to the polymeric matrix, high measurements were resulted compared to the neat PLC film, and hence, this implies both drug-loaded films are more hydrophobic than the neat polymer film. A drug solubility test in the relatively polar solvents (DCM and THF) was also conducted to determine loading limits for film casting. LFX solubility in DCM and THF was found to be 68 mg/ml and 27.9 mg/ml respectively; while MFX presented lower solubility of 0.42 mg/ml in DCM and 0.016 mg/ml in THF; this reflects the relative hydrophobicities of the drug as measured via contact angle analysis.

In the first study phase, the drug release performance of both antibiotics (from cast films) was investigated for 14 days according to the treatment period. A biphasic release was observed from LFX and MFX loaded films. The rapid release of drug observed from LFX-DCM films was due to the drug segregated at or near the surface of the films. It was postulated that the rapid evaporation of volatile DCM caused severe phase separation of drug particles from the polymer matrix to migrate and aggregate on the surface [31]. This postulate was supported by the SEM images of the films (Fig 4).

In general, DCM tends to form surface morphologies that are highly porous as compared to samples fabricated using THF. This is particularly evident from the SEM micrographs of LFX films. The highly porous morphologies from LFX-DCM resulted in a huge initial burst release and by changing the solvent to THF, a significant reduction in the burst release was observed. The suppression of the initial burst and subsequent release is related to the reduced surface porosity on the films (Fig 5). Hence, a sustained release could be obtained.

MFX films, on the other hand, showed similar morphologies with both solvents. As a result, the burst releases of MFX from films cast from either solvent are not substantially different (Table 1). At higher loadings of MFX in either solvent, the burst is higher, in keeping with expectation that the dispersed drug (above the solubility limit in the polymer) releases in a burst from the surface. Therefore, this shows a correlation of the release behavior with the observed hydrophobicity of the films. To summarize the observations from the film studies, therefore:

1. LFX-loaded films at 15, 20, 25% cast from DCM all show substantial burst release with complete release seen in 24–48 hours. This is due to significant porosity and loading of drug above its solubility limit in the polymer matrix.
2. Changing the solvent to THF decreases the burst significantly for MFX and for LFX; the surface scan shows very little porosity, but extent of burst is proportional to loading, as expected.
3. Based on these data, THF was chosen as the solvent to be used in the attachment, and both drugs were evaluated at different loadings.

**Table 1:** Summary of burst release from all films.

	Burst Release		
	Films		
Formulations	15%	20%	25%
LFX (DCM)	96.0 ± 2.1%	97.8 ± 2.4%	97.7 ± 0.4%
LFX (THF)	18.5 ± 2.1%	17.3 ± 0.7%	33.0 ± 1.7%
MFX (DCM)	5.6 ± 1.1%	12.9 ± 1.3%	15.5 ± 2.5%
MFX (THF)	7.4 ± 0.3%	14.4 ± 0.9%	27.1 ± 1.6%

THF film formulations that achieved sustained release were selected for IOL attachment fabrication. Ultrasonic spray coated attachments yielded smooth and uniform surfaces. However, high drug-loaded LFX formulations exhibited a higher burst release than was expected from the film studies (Table 2). This is primarily due to the larger surface area-to-volume ratio of the attachments. Typically, this ratio is about 102.8 for the attachment and 81.3 for the films. As all the higher drug loadings studied initially (15, 20 and 25%) were above the solubility limit of drug in matrix, the burst release was along expected lines. A more sustained-release effect was only observed after lowering the drug loading to 5% and to 3%. In general, the higher the drug loading, the higher the extent of burst release; thus implying a low saturation limit (~3%) of the drug (LFX in this case) in the polymer. The drug amounts released daily from attachments were also lesser compared to films.

**Table 2:** Summary of burst release from LFX and MFX attachments.

	Burst Release				
	Coatings				
	3%	5%	15%	20%	25%
LFX (THF)	14.7 ± 0.7%	51.6 ± 7.1%	-	78.4 ± 1.0%	68.6 ± 3.8%
MFX (THF)	-	-	1.0 ± 0.3%	1.0 ± 0.6%	1.9 ± 1.1%

This difference between films and attachments was seen likewise for MFX. Although the higher drug loadings in MFX films were above the solubility limit of drug in matrix, drug molecules were entrapped between film layers. However in the case of attachments, such high drug loaded solutions could not be effectively coated onto the mandrel. Therefore, the attachments suffered from drug distribution non-uniformity and overall low drug release, leading to large error bars in quantification. The analysis method using HPLC to quantify drug is specific to the molecular configuration or structure of the drug, hence it can be indirectly concluded that the drug released are stable and in its active form. An estimate shows that the MIC value against S. Aureus for Levofloxacin is 2 μg/mL and for Moxifloxacin is 0.5 μg/mL. Considering the aqueous volume in humans to be 250 μL, the average daily release from films i.e. 15% LFX-loaded and MFX-loaded films are estimated to be 160 μg/mL and 32 μg/mL, respectively. For LFX coatings, the minimum average daily release is approximately 8 μg/mL even on day 14 of the release study. For MFX, the minimum average daily release is approximately 1.6 μg/mL. Both the films and coatings demonstrated higher concentration than the inhibitory concentration and hence, it can be postulated that both systems would be effective against S.Aureus. However, the author recommends further follow up studies using specific bio-assay for evaluation of drug bioactivity of released drug, antimicrobial and long term cell adhesion test.

In summary, the spray-coating technique was able to generate attachments with loadings of 3% and 5% LFX, from THF solutions; such attachments were able to achieve a 2-week sustained release within the therapeutic window.

## Conclusion

In this study, a novel drug-eluting attachment to IOLs was designed and developed. The selection of solvent is also important: our cast film studies showed that a slower-evaporating solvent is to be preferred for spray-coating, because it leads to more uniform films with little porosity. Based on in vitro results of the effects of drug loading, solvent and mode of fabrication on drug release behavior, we have selected THF as the optimal solvent and the optimum IOL-attachment composition was between 3 to 5% of LFX in a biodegradable PLC matrix. Such a drug-eluting attachment to the IOL can release LFX at the required therapeutic doses over 14 days, and hence is an attractive option to overcome issues in post-IOL implantation, such as infections and the poor patient compliance seen with eye drops.
